# Operationalisation of post‐COVID condition case definition in a comprehensive research protocol

**DOI:** 10.1111/ene.16543

**Published:** 2024-11-13

**Authors:** Federico Masserini, Alessia Nicotra, Arianna Forgione, Francesca Calcaterra, Elena Perdixi, Clara Di Vito, Anna Carletti, Chiara Gallo, Pietro Emiliano Doneddu, Fabrizia Terenghi, Simone Pomati, Eduardo Nobile‐Orazio, Agostino Riva, Domenico Mavilio, Leonardo Pantoni

**Affiliations:** ^1^ Department of Biomedical and Clinical Sciences, Neuroscience Research Center University of Milan Milan Italy; ^2^ Unit of Clinical and Experimental Immunology IRCCS Humanitas Research Hospital Milan Italy; ^3^ Department of Medical Biotechnologies and Translational Medicine (BioMeTra) University of Milan Milan Italy; ^4^ Department of Neurology IRCCS Humanitas Research Hospital Milan Italy; ^5^ Neuromuscular and Neuroimmunology Unit IRCCS Humanitas Research Hospital Milan Italy; ^6^ Department of Biomedical Sciences Humanitas University Milan Italy

**Keywords:** case definition, cognitive, COVID‐19, post‐COVID condition, psychiatric

## Abstract

**Background and purpose:**

Post‐COVID‐19 condition (PCC) is a prevalent and high‐burden sequela of SARS‐CoV‐2 infection. Because of the complexity of its manifestations, PCC case definition currently lacks standardisation and reproducibility. We aimed to devise a simple screening tool to boost reproducibility and comparability of PCC case definition across PCC studies, and to provide a framework in which to reliably identify suspected PCC cases. We also developed a comprehensive assessment protocol based on the most frequently reported PCC characteristics.

**Methods:**

Within a European multi‐centre study and based on the conclusions of a previous systematic review, we devised a checklist to assess the presence of PCC‐associated symptoms and their temporal relationship with COVID‐19.

**Results:**

The checklist was developed to include three symptom cores (cognitive, psychiatric, and general). For each symptom, onset is assessed within 3 months after COVID‐19 resolution and persistence for at least 2 months. Any symptom fulfilling this criterion is sufficient to prompt suspicion of PCC. At least one symptom is required in the cognitive or psychiatric domains to suspect PCC with neuropsychiatric involvement. Our protocol features an extensive neuropsychological evaluation and self‐administered scales for mood, anxiety, stress‐related symptoms, sleep disorders, quality of life, disability, mental health, and personality traits; scales for quantitative assessment of fatigue and headache are also included.

**Conclusions:**

Consistent identification of PCC cases is crucial to correctly include patients in research and clinical studies. We propose a simple, reproducible, and flexible screening tool and a proposal for a comprehensive assessment that could be employed to enhance standardisation and comparability.

## INTRODUCTION

Post‐COVID condition (PCC) is one of the most prevalent complications of SARS‐CoV‐2 infection, especially in non‐hospitalised patients [1]. However, PCC case definition is broad and non‐standardised. For this reason, the precise identification of patients affected by this condition and the establishment of a pathophysiological link with primary SARS‐CoV‐2 infection are still difficult. Attempts to devise reliable diagnostic criteria for PCC have been limited by the number and breadth of its manifestations [2]. Accordingly, the most recent proposals by the World Health Organisation (WHO) and the National Institute for Health and Care Excellence (NICE) [3, 4] provide only a timeframe for symptom onset and persistence, without circumscribing PCC‐associated manifestations. In addition to the lack of a standardised definition, we also lack common protocols for patient assessment, and different studies have almost invariably used assessment protocols that differ both in scope of assessment and single tests employed.

Despite the overwhelming number of described symptoms, several recurring key manifestations of PCC have emerged, as shown in a recent systematic review [5]. These manifestations include cognitive (e.g., memory/attention difficulties, and brain fog), psychiatric (e.g., mood disorders, anxiety, stress) and ‘systemic/general’ symptoms (e.g., fatigue, headache), and likely constitute a common ground for a PCC definition. Peripheral nervous system symptoms and dysautonomia have also been reported; however, their prevalence in PCC is unclear, as is their co‐occurrence with more frequent symptoms [6]. Moreover, a significant proportion of these may be direct consequences of acute critical illness or part of established post‐infectious sequelae independent of PCC (e.g., acute‐immune polyradiculoneuropathy).

Laying the groundwork for a widely shared case definition of PCC is paramount to ensure consistent participant enrolment in observational studies, to foster comparability, and ultimately to improve the chances of understanding the pathophysiological underpinnings of this condition [7]. Moreover, given the breadth of its cognitive and psychiatric manifestations, we advocate also for the focused, comprehensive neuropsychological and psychiatric symptom evaluation of PCC [5, 8, 9, 10].

Within a European multi‐centre observational cohort study (Horizon Project ID n.101057775), we aimed to create a simple and flexible instrument to operationalise PCC case definition, in an attempt to create a common framework for PCC screening. Furthermore, based on the most commonly associated symptoms and findings, we propose a comprehensive assessment protocol that could be adopted in clinical and research settings.

## METHODS

We created a checklist that includes the most prevalent co‐occurring features reported in PCC, which have emerged from a systematic review as well as from clinical experience [5, 10]. This checklist is designed to assess whether each symptom is present and whether its timeframe of onset and persistence fulfils WHO criteria, to assess for plausible association with primary SARS‐CoV‐2 infection. Based on checklist assessment, we also provided a criterion to define possible PCC cases. Other possibly relevant symptoms, whose association with PCC is less clear or established, were reported in a checklist (supplementary appendix—Data S1) for further phenotyping only (not for PCC case definition). Finally, considering target population characteristics, the most frequently reported symptoms, and reported alterations in cognitive/psychiatric domains, we devised a comprehensive protocol for cognitive, psychiatric and general symptom evaluation.

## RESULTS

We divided symptoms included in the checklist into three core domains: cognitive, psychiatric, and ‘systemic/general’ cores. For each symptom, the checklist requires that the symptom is a new‐onset symptom (i.e., it was not present before COVID‐19) and that it fulfils the WHO onset/persistence requirements, namely, (i) it began within 3 months from acute infection resolution (i.e., from the date of the first negative nasopharyngeal swab or, if not available, from the first positive nasopharyngeal swab) and (ii) it has persisted for at least 2 months after onset.

**FIGURE 1:**
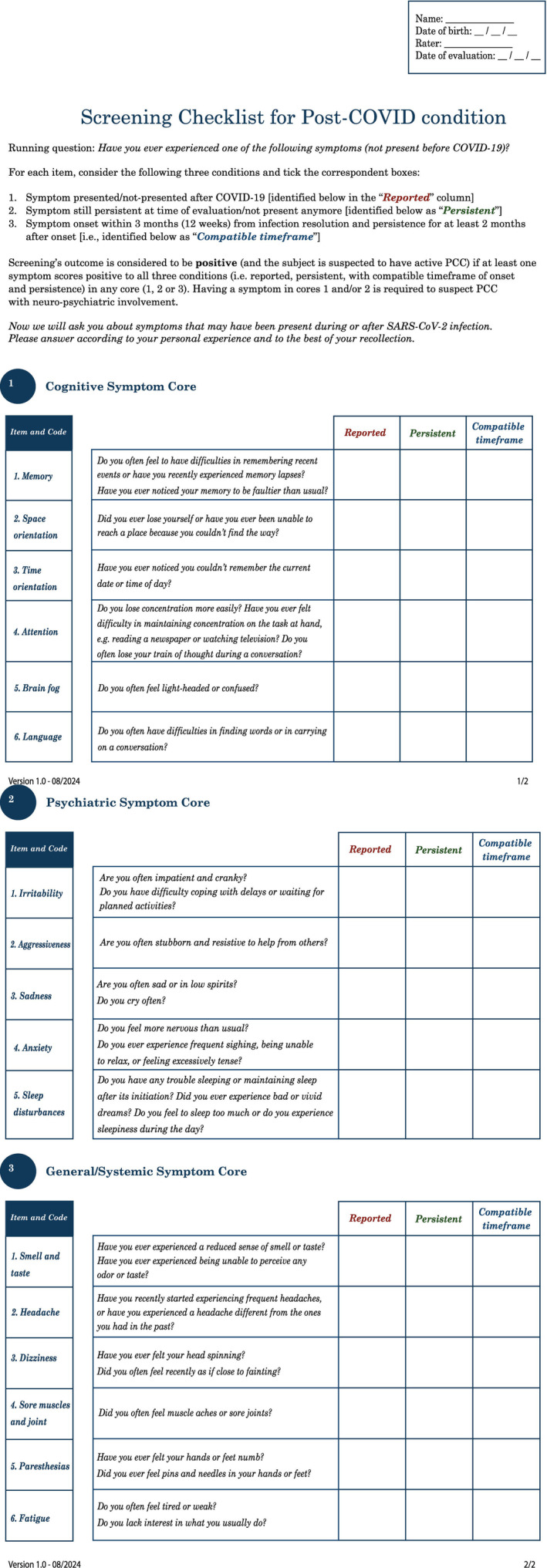
Complete symptom checklist, along with instruction for administration. Questions are phrased in plain language and are intended for direct administration (also self‐administration if deemed appropriate). PCC, post‐COVID condition.

Any single symptom fulfilling all the above‐mentioned requirements is sufficient to suspect PCC. The presence of at least one symptom belonging to the cognitive or psychiatric cores is required to define PCC with cognitive/psychiatric involvement. The checklist is reported in Figure 1, while its supplementary appendix, containing other possibly relevant symptoms, is reported in the Supplementary Materials (eFigure 1— Data S1, *a printable version of the checklist is available in the Supplementary Materials ‐ Data* S3). A web‐based publicly available version of the tool (codename: screen‐PCC) is available in the RedCap shared library (https://redcap.vanderbilt.edu/consortium/library/search.php).

**FIGURE 2:**
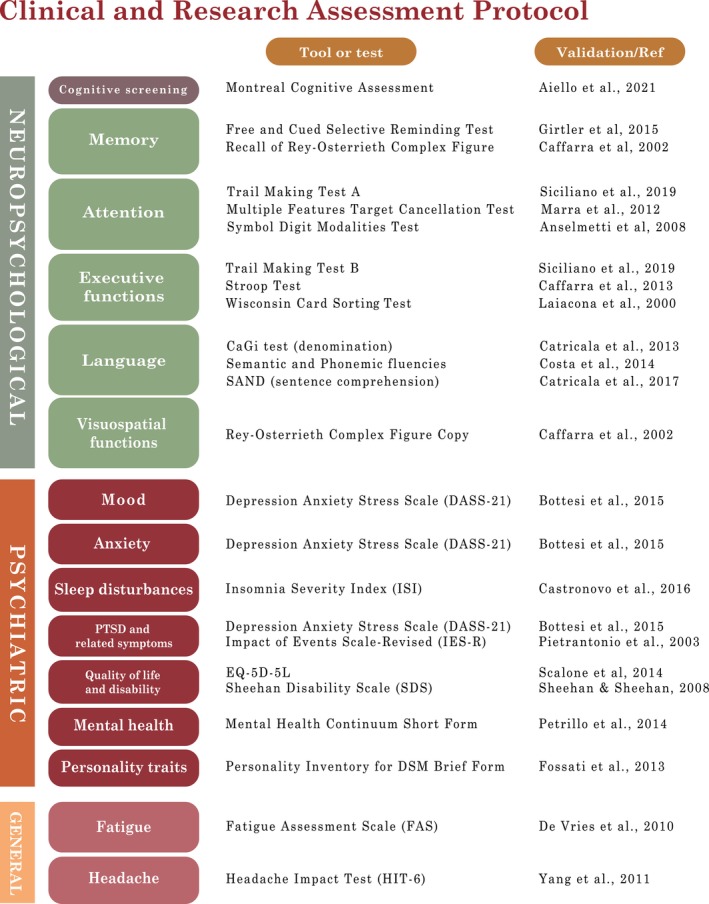
Outline of the clinical and research assessment protocol. A complete list of neuropsychological tests, psychiatric/behavioral scales and symptom scale are reported, along with pertinent references or validation studies in the Italian population, if available. DSM, Diagnostic and Statistical Manual of Mental Disorders; PTSD, post‐traumatic stress disorder; Ref, reference (the complete references are reported in the Supplementary Materials ‐ Data S2).

Within the European study, we devised a protocol for patient assessment that features cognitive, psychiatric and other relevant general symptom evaluation (as outlined in Figure 2). Cognitive assessment consists of a screening test (Montreal Cognitive Assessment) and a comprehensive neuropsychological test battery. Psychiatric symptom evaluation features self‐administered scales for mood disorders, anxiety, stress symptoms, sleep disturbance, quality of life, and disability. The battery also includes assessments of mental health and personality traits. General symptoms, such as fatigue and new‐onset headache, are also assessed and graded. A list of the instruments used for each area of evaluation and validation/normative studies for the general population is reported in Figure 2 and in the Supplementary Materials—Data S2.

## DISCUSSION

To operationalise the case definition that emerged from our systematic review on PCC [5], we developed a tool to screen suspected PCC cases that integrates an evidence‐based symptom checklist and the assessment of the temporal relationship with primary infection, as suggested by NICE and the WHO. We wanted to make this tool sufficiently simple and rapid to administer in order to boost its adoption and enhance administration reliability.

We believe a shared definition, rooted in evidence, may enhance not only comparability across studies but also the reliability of case definition itself. This, in turn, increases the chances of study success, as it facilitates reliable and reproducible discrimination between PCC cases and non‐PCC controls.

Since PCC comprises several non‐specific symptoms, and because the biological relationship with COVID‐19 has still to be demonstrated for most of these, we decided to propose a broad sensitive criterion (a single symptom is sufficient to classify the participant as a PCC case) that is mainly intended to raise PCC suspicion (without providing any degree of certainty to the diagnosis). This is crucial because, at this time, we cannot distinguish symptoms that are likely to be underpinned by SARS‐CoV‐2 infection from manifestations that are possibly associated with intervening and/or pre‐existing psychosocial factors [8, 11, 12]. For these same reasons, it is important to have a screening tool that is not only useful for raising suspicion of PCC diagnosis but has also been devised to collect data to further stratify our patients in the future. Accordingly, our screening tool is built with flexibility in mind, as symptoms may be added or removed from the checklist based on evidence that may become available in the future. Indeed, we have already provided a checklist appendix to score symptoms whose relevance to the syndrome is still to be reliably and consecutively assessed.

According to the same evidence‐based approach used to build the checklist, we devised a comprehensive assessment protocol to objectively assess PCC manifestations. For the neuropsychological evaluation, we chose a battery of tests that encompasses all major cognitive domains, with particular focus on the most frequently affected domains (i.e., attentive/executive domains and memory), and is tailored to the young and highly educated population that is typically affected by PCC [10, 12]. Recognising the high impact of this condition on patient's everyday life [13, 14] and the reported role of psychosocial factors, we also chose to add measures of quality of life, disability, and mental health. Finally, we chose to include symptom scales to estimate the quantitative burden of two of the most common general manifestations [3, 5].

We must acknowledge that our symptom checklist is not exhaustive, especially as far as general/systemic symptoms are concerned. However, at this stage, we believe that limiting the number of assessed symptoms to those most commonly associated with PCC may be a sensible approach to reduce complexity and boost the chance of study success. We recognise that lack of translation or validation of some of the tests in different countries may limit the immediate applicability of our protocol and that its length may limit applicability in clinical settings. Nonetheless, the same general principles that constitute our assessment protocol foundation could be used to guide the choice of alternative tests that are validated in or more suitable for other target populations, as well as to devise shorter test batteries more suited for clinical use.

Our definition differs in approach from that recently published by the National Academies of Sciences, Engineering, and Medicine (NASEM) [15]. While the former was created with inclusivity and the broadest possible clinical setting in mind, to ensure no patient with lingering sequelae after COVID‐19 is left out, ours was derived mainly within a research‐oriented clinical setting, to boost reproducibility and hence our chances of elucidating pathophysiology and possibly pave a way to treatment. Accordingly, its scope is specifically restricted to PCC rather than ‘long‐COVID’, an umbrella term with a loose meaning, used to refer to any manifestation occurring after COVID‐19. Furthermore, while the NASEM definition does not require a timeframe from symptom onset after COVID‐19, our temporal boundaries are stricter and abide by the widely used WHO definition, to increase chances of plausible association with acute infection.

Considering the limited comparability across studies in PCC caused by the different definitions used, we propose a simple, flexible and reproducible tool to assess and screen participants suspected of PCC, to provide an operative case definition that may enhance comparability and reproducibility. Currently, this tool is mainly aimed at raising PCC suspicion and at selecting subjects for inclusion in research studies. Further studies are needed to increase our understanding of this complex condition and to assess the specificity of our criteria.

## AUTHOR CONTRIBUTIONS

**Federico Masserini:** Conceptualization; methodology; software; writing – original draft; writing – review and editing; supervision; investigation. **Alessia Nicotra:** Conceptualization; methodology; writing – review and editing; investigation. **Arianna Forgione:** Conceptualization; methodology; writing – review and editing; investigation. **Francesca Calcaterra:** Conceptualization; writing – review and editing; investigation. **Elena Perdixi:** Conceptualization; writing – review and editing; investigation. **Clara Di Vito:** Conceptualization; writing – review and editing; investigation. **Anna Carletti:** Investigation; methodology; writing – review and editing. **Chiara Gallo:** Investigation; writing – review and editing. **Pietro Emiliano Doneddu:** Writing – review and editing; conceptualization; methodology. **Fabrizia Terenghi:** Conceptualization; writing – review and editing; investigation. **Simone Pomati:** Conceptualization; writing – review and editing. **Eduardo Nobile‐Orazio:** Conceptualization; writing – review and editing. **Agostino Riva:** Conceptualization; methodology; investigation; writing – review and editing; supervision. **Domenico Mavilio:** Conceptualization; supervision; writing – review and editing. **Leonardo Pantoni:** Conceptualization; methodology; supervision; writing – review and editing; writing – original draft.

## FUNDING INFORMATION

This project has received funding from the RIA HORIZON Research and Innovation Actions under GA no. 101057775, funded by the European Union. The views and opinions expressed, however, are those of the author(s) only and do not necessarily reflect those of the European Union or European Health and Digital Agency (HADEA). Neither the European Union nor the granting authority can be held responsible for them.

## CONFLICT OF INTEREST STATEMENT

The authors declare no conflict of interest.

## Supporting information


**Data S1.** Supporting information.

**Data S2.** Supporting information.

**Data S3.** Supporting information.

## Data Availability

Data sharing not applicable to this article as no datasets were generated or analysed during the current study.
