# Supplementary material for: Operationalisation of post‐COVID condition case definition in a comprehensive research protocol
Source: Eur J Neurol. 2024 Nov 13;32(1):e16543. doi: 10.1111/ene.16543 (PMC11625920; doi:10.1111/ene.16543)
Supplement: Supplementary file 1 — Data S1. Supporting information. [file ENE-32-e16543-s001.pdf]

Name: ______________
Date of birth: __ / __ / __
Rater: ______________
Date of evaluation: __ / __ / __

Screening Checklist for Post-COVID condition

Supplementary appendix

Running question: *Have you ever experienced one of the following symptoms (not present before COVID-19)?*

For each item, consider the following three conditions and tick the correspondent boxes:

1. Symptom presented/not-presented after COVID-19 [identified below in the "***Reported***" column]
2. Symptom still persistent at time of evaluation/not present anymore [identified below as "***Persistent***"]
3. Symptom onset within 3 months (12 weeks) from infection resolution and persistence for at least 2 months after onset [i.e., identified below as "***Compatible timeframe***"]

Screening of symptoms reported in this appendix is currently considered only for further phenotyping and stratification. Presence of these symptoms alone (i.e. in absence of other symptoms reported in the main checklist cores) should not be employed to suspect Post-COVID condition.

Now we will ask you about symptoms that may have been present during or after SARS-CoV-2 infection. Please answer according to your personal experience and to the best of your recollection.

A Peripheral/autonomic nervous system symptoms

Item and Code		*Reported*	*Persistent*	*Compatible timeframe*
1. Paresthesias	*Do you feel your hands/of feet as numb or tingling persistently (i.e., present every day, all day long)?*			
2. Dysesthesias	*Do you perceive a burning sensation or electric shock-like feeling in the hands and/or feet persistently? (i.e., present every day, all day long).*			
3. Hyposthenia	*Have you experienced the onset of significant weakness in the hands or legs (such as difficulty walking, climbing stairs, opening water bottles, or turning a key in a lock) persistent to the present day (present every day, all day long)?*			
4. Postural instability	*Have you experienced persistent postural instability (present every day, all day long)? Have you experienced frequent falls?*			
5. Other visceral	*Do your mouth feel persistently dry? Have you experienced recurring episodes of excessive sweating, diarrhea, chronic constipation, or frequent fainting? (episodes must be frequent, i.e. symptoms must be present almost every day).*			
