# Supplementary material for: Operationalisation of post‐COVID condition case definition in a comprehensive research protocol
Source: Eur J Neurol. 2024 Nov 13;32(1):e16543. doi: 10.1111/ene.16543 (PMC11625920; doi:10.1111/ene.16543)
Supplement: Supplementary file 2 — Data S2. Supporting information. [file ENE-32-e16543-s002.docx]

**Supplementary materials**

Supplementary References (extended Figure 2 references):

1. Aiello EN, Gramegna C, Esposito A, et al. The Montreal Cognitive Assessment (MoCA): updated norms and psychometric insights into adaptive testing from healthy individuals in Northern Italy. *Aging Clinical and Experimental Research*. 2021;34(2):375-382. doi:10.1007/s40520-021-01943-7

2. Anselmetti S, Poletti S, Ermoli E, et al. The Brief Assessment of Cognition in Schizophrenia. Normative data for the Italian population. *Neurol Sci*. Apr 2008;29(2):85-92. doi:10.1007/s10072-008-0866-9

3. Bottesi G, Ghisi M, Altoe G, Conforti E, Melli G, Sica C. The Italian version of the Depression Anxiety Stress Scales-21: Factor structure and psychometric properties on community and clinical samples. *Compr Psychiatry*. Jul 2015;60:170-81. doi:10.1016/j.comppsych.2015.04.005

4. Caffarra P, Vezzadini G, Dieci F, Zonato F, Venneri A. Rey-Osterrieth complex figure: normative values in an Italian population sample. *Neurol Sci*. Mar 2002;22(6):443-7. doi:10.1007/s100720200003

5. Caffarra P, Vezzadini G, Dieci F, Zonato F, Venneri A. A short version of the Stroop test: Normative data in an Italian population sample. Article. *Nuova Rivista di Neurologia*. 2002;12(4):111-115.

6. Castronovo V, Galbiati A, Marelli S, et al. Validation study of the Italian version of the Insomnia Severity Index (ISI). *Neurol Sci*. Sep 2016;37(9):1517-24. doi:10.1007/s10072-016-2620-z

7. Catricala E, Della Rosa PA, Ginex V, Mussetti Z, Plebani V, Cappa SF. An Italian battery for the assessment of semantic memory disorders. *Neurol Sci*. Jun 2013;34(6):985-93. doi:10.1007/s10072-012-1181-z

8. Catricala E, Gobbi E, Battista P, et al. SAND: a Screening for Aphasia in NeuroDegeneration. Development and normative data. *Neurol Sci*. Aug 2017;38(8):1469-1483. doi:10.1007/s10072-017-3001-y

9. Costa A, Bagoj E, Monaco M, et al. Standardization and normative data obtained in the Italian population for a new verbal fluency instrument, the phonemic/semantic alternate fluency test. *Neurol Sci*. Mar 2014;35(3):365-72. doi:10.1007/s10072-013-1520-8

10. de Vries J, Michielsen H, Van Heck GL, Drent M. Measuring fatigue in sarcoidosis: The Fatigue Assessment Scale (FAS). *British Journal of Health Psychology*. 2010;9(3):279-291. doi:10.1348/1359107041557048

11. Fossati A, Krueger RF, Markon KE, Borroni S, Maffei C. Reliability and validity of the personality inventory for DSM-5 (PID-5): predicting DSM-IV personality disorders and psychopathy in community-dwelling Italian adults. *Assessment*. Dec 2013;20(6):689-708. doi:10.1177/1073191113504984

12. Girtler N, De Carli F, Amore M, et al. A normative study of the Italian printed word version of the free and cued selective reminding test. *Neurol Sci*. Jul 2015;36(7):1127-34. doi:10.1007/s10072-015-2237-7

13. Marra C, Gainotti G, Scaricamazza E, Piccininni C, Ferraccioli M, Quaranta D. The Multiple Features Target Cancellation (MFTC): an attentional visual conjunction search test. Normative values for the Italian population. *Neurological Sciences*. 2012;34(2):173-180. doi:10.1007/s10072-012-0975-3

14. Petrillo G, Capone V, Caso D, Keyes CLM. The Mental Health Continuum–Short Form (MHC–SF) as a Measure of Well-Being in the Italian Context. *Social Indicators Research*. 2014;121(1):291-312. doi:10.1007/s11205-014-0629-3

15. Pietrantonio F, De Gennaro L, Di Paolo MC, Solano L. The Impact of Event Scale: validation of an Italian version. *J Psychosom Res*. Oct 2003;55(4):389-93. doi:10.1016/s0022-3999(02)00638-4

16. Scalone L, Cortesi PA, Mantovani LG, Ciampichini R, Cesana G. Reference Eq-5d-3l and Eq-5d-5l Data From the Italian General Population. *Value Health*. Nov 2014;17(7):A514-5. doi:10.1016/j.jval.2014.08.1591

17. Sheehan KH, Sheehan DV. Assessing treatment effects in clinical trials with the discan metric of the Sheehan Disability Scale. *Int Clin Psychopharmacol*. Mar 2008;23(2):70-83. doi:10.1097/YIC.0b013e3282f2b4d6

18. Siciliano M, Chiorri C, Battini V, et al. Regression-based normative data and equivalent scores for Trail Making Test (TMT): an updated Italian normative study. *Neurol Sci*. Mar 2019;40(3):469-477. doi:10.1007/s10072-018-3673-y

19. Yang M, Rendas-Baum R, Varon SF, Kosinski M. Validation of the Headache Impact Test (HIT-6) across episodic and chronic migraine. *Cephalalgia*. Feb 2011;31(3):357-67. doi:10.1177/0333102410379890

Supplementary Figures legends:

**eFigure 1***:* Symptoms checklist supplementary appendix featuring peripheral/autonomic nervous system symptoms (intended for further patient phenotyping only, not for PCC case definition). Questions are phrased in plain language and are thought for direct administration (also self-administration if deemed appropriate).
