# Supplementary material for: Operationalisation of post‐COVID condition case definition in a comprehensive research protocol
Source: Eur J Neurol. 2024 Nov 13;32(1):e16543. doi: 10.1111/ene.16543 (PMC11625920; doi:10.1111/ene.16543)
Supplement: Supplementary file 3 — Data S3. Supporting information. [file ENE-32-e16543-s003.pdf]

Name: ______________
Date of birth: __ / __ / __
Rater: ______________
Date of evaluation: __ / __ / __

Screening Checklist for Post-COVID condition

Running question: *Have you ever experienced one of the following symptoms (not present before COVID-19)?*

For each item, consider the following three conditions and tick the correspondent boxes:

1. Symptom presented/not-presented after COVID-19 [identified below in the "***Reported***" column]
2. Symptom still persistent at time of evaluation/not present anymore [identified below as "***Persistent***"]
3. Symptom onset within 3 months (12 weeks) from infection resolution and persistence for at least 2 months after onset [i.e., identified below as "***Compatible timeframe***"]

Screening's outcome is considered to be **positive** (and the subject is suspected to have active PCC) if at least one symptom scores positive to all three conditions (i.e. reported, persistent, with compatible timeframe of onset and persistence) in any core (1, 2 or 3). Having a symptom in cores 1 and/or 2 is required to suspect PCC with neuro-psychiatric involvement.

Now we will ask you about symptoms that may have been present during or after SARS-CoV-2 infection. Please answer according to your personal experience and to the best of your recollection.

1 Cognitive Symptom Core

Item and Code		*Reported*	*Persistent*	*Compatible timeframe*
1. Memory	*Do you often feel to have difficulties in remembering recent events or have you recently experienced memory lapses? Have you ever noticed your memory to be faultier than usual?*			
2. Space orientation	*Did you ever lose yourself or have you ever been unable to reach a place because you couldn't find the way?*			
3. Time orientation	*Have you ever noticed you couldn't remember the current date or time of day?*			
4. Attention	*Do you lose concentration more easily? Have you ever felt difficulty in maintaining concentration on the task at hand, e.g. reading a newspaper or watching television? Do you often lose your train of thought during a conversation?*			
5. Brain fog	*Do you often feel light-headed or confused?*			
6. Language	*Do you often have difficulties in finding words or in carrying on a conversation?*			

2 Psychiatric Symptom Core

Item and Code		*Reported*	*Persistent*	*Compatible timeframe*
1. Irritability	*Are you often impatient and cranky?*<br>*Do you have difficulty coping with delays or waiting for planned activities?*			
2. Aggressiveness	*Are you often stubborn and resistive to help from others?*			
3. Sadness	*Are you often sad or in low spirits?*<br>*Do you cry often?*			
4. Anxiety	*Do you feel more nervous than usual?*<br>*Do you ever experience frequent sighing, being unable to relax, or feeling excessively tense?*			
5. Sleep disturbances	*Do you have any trouble sleeping or maintaining sleep after its initiation? Did you ever experience bad or vivid dreams? Do you feel to sleep too much or do you experience sleepiness during the day?*			

3 General/Systemic Symptom Core

Item and Code		*Reported*	*Persistent*	*Compatible timeframe*
1. Smell and taste	*Have you ever experienced a reduced sense of smell or taste?*<br>*Have you ever experienced being unable to perceive any odor or taste?*			
2. Headache	*Have you recently started experiencing frequent headaches, or have you experienced a headache different from the ones you had in the past?*			
3. Dizziness	*Have you ever felt your head spinning?*<br>*Did you often feel recently as if close to fainting?*			
4. Sore muscles and joint	*Did you often feel muscle aches or sore joints?*			
5. Paresthesias	*Have you ever felt your hands or feet numb?*<br>*Did you ever feel pins and needles in your hands or feet?*			
6. Fatigue	*Do you often feel tired or weak?*<br>*Do you lack interest in what you usually do?*			
